# *Astilbe uljinensis* (Saxifragaceae), a new species from South Korea

**DOI:** 10.3897/phytokeys.161.53019

**Published:** 2020-09-29

**Authors:** Ami Oh, Sea-Hee Han, Byoung-Un Oh

**Affiliations:** 1 School of Biological Sciences, Chungbuk National University, Cheongju 28644, South Korea Chungbuk National University Cheongju South Korea; 2 National Agrobiodiversity Center, National Academy of Agricultural Science RDA, Jeonju 54875, South Korea National Agrobiodiversity Center Jeonju South Korea

**Keywords:** Gangwon-do, Gyeongsangbuk-do, South Korean endemic, morphology, taxonomy, trichome

## Abstract

A new species *Astilbe uljinensis* B.U.Oh & H.J.Choi is described from Gangwon-do and Kyeongsangbuk-do in South Korea based on its morphological characteristics and distributional pattern. *A. uljinensis* is easily distinguished not only from three other *Astilbe* species in South Korea, but all other species in the genus by possessing a green young rachis, dense long whitish glandular hairs on the young rachis, dense long brownish glandular hairs on the mature inflorescence, and a slightly undulated margin of leaf epidermal cells. Specific comparisons of morphological features such as the type of the trichome, the shape of the leaf epidermis cell, and the color of the young rachis that differentiate *A. uljinensis* from *Astilbe chinensis*, another South Korean *Astilbe* species, are provided.

## Introduction

*Astilbe* Buchanan-Hamilton. & D. Don (Saxifragaceae, Saxifragales) is composed of approximately 18 species which are distributed in eastern and southern Asia, and eastern North America (Pan 1985). Here, eastern and southern Asian regions include Russia, India, Bhutan, Nepal, Thailand, Indonesia, Philippines, South Korea, China, and Japan. The distributional pattern of the genus exhibits a clear geographical disjunction between eastern Asia and eastern North America (Li 1952; Pan 1995; Kim et al. 2009; Zhu et al. 2013).

Owing to the large morphological variations in *Astilbe*, the taxonomy of South Korean *Astilbe* has remained incomplete to date (Chung 1957; Lee 1980; Lee 1996a, b; Im 1997).Taxonomic studies on South Korean *Astilbe* species were first conducted by Chung et al. (1983) and Kim (1987), who recorded 5 species and 3 varieties, and 1 species and 3 varieties, respectively. In illustrated books and directory books, Lee (1980) recorded 2 species and 1 variety, Lee (1996a) recorded 3 species and 2 varieties, Lee (1996b) recorded 2 species, 2 varieties, and 1 form, while Im (1997) recorded 1 species and 4 varieties. Subsequently, Kim (2007) recorded 2 species and 2 varieties. Recently, in South Korea, three *Astilbe* species, including *Astilbe chinensis*, *Astilbe taquetii*, and *Astilbe koreana*, were unambiguously recognized (Han and Oh 2019).

In this study, we report a previously undescribed species whose morphology is clearly distinct from all other species of *Astilbe*. This undescribed species is restricted to Kyeongsangbuk-do (Uljin-gun) and Gangwon-do (Gangneung-si, Pyeongchang-gun, Samcheok-si), the central regions of South Korea, particularly on the eastern and southern sides of the Baekdudaegan mountain ranges near the eastern coast. Based on morphological and distributional characteristics, this species is identified and described as a novel species from South Korea.

## Materials and methods

For species description, the type materials in the herbarium of Chungbuk National University (**CBU**), which were collected by the authors between May 2013 and September 2015, were examined. The vouchers in the herbarium of Korean National Arboretum (**KH**) and the herbarium of Kangwon National University (**KWNU**) were also investigated. In addition, the specimens were investigated extensively in the wild, and some of the collected plants of the novel species were planted in the cultivation facility of Chungbuk National University for the study of their morphology. Between 10 and 30 individuals were used in the quantitative morphological feature measurements.

A comparison with a related taxon was made against *Astilbe chinensis*, one of the South Korean *Astilbe* species that is morphologically most similar to the newly described species. *A. chinensis* is distributed in the East Asian countries of South Korea, China, Russia and Japan (Pan and Ohba 2001; Han and Oh 2019). In some previous studies, this species was incorrectly considered as a synonym of *Astilbe rubra*, which is distributed in China and India (Lee 1996a; Ohba 2001; Kim 2007; Kim et al. 2009). The vouchers of *A. chinensis* in the herbarium CBU and the living plants in the cultivation facility at Chungbuk National University were examined for the comparison. A previous study on South Korean *Astilbe* species (Han and Oh 2019) was also referred to for the information on *A. chinensis*.

The morphological observations throughout this study were performed using Zoom 2000 (Leica, Germany), a stereoscopic microscope, ECLIPSE E600 (Nikon, Japan), a light microscope, and JSM-700F (Jeol, Japan), a scanning electron microscope. The brownish glandular hairs on the inflorescence and the whitish glandular hairs on the young shoots were observed using the stereoscopic microscope, and the epidermal cells and the stomatal apparatus were observed using the light microscope and the scanning electron microscope.

## Taxonomic treatment

### Astilbe uljinensis

Taxon classificationPlantaeSaxifragalesSaxifragaceae

B.U.Oh & H.J.Choi sp. nov.

BFDD4012-F6B2-596F-89FD-44F2A5336E9F

urn:lsid:ipni.org:names:77211769-1

Figs 1 , 2

#### Diagnosis.

This new species is similar to *A. chinensis*, but differs from it due to the presence of a green young rachis of rhizomatous leaf, dense whitish long glandular hairs and sparse brownish long non-glandular hairs on the young rachis, dense brownish long glandular hairs of 1.5 to 2.7 mm length on the mature inflorescence, ovate-elliptic terminal leaflet, weakly undulated margin of leaf adaxial epidermal cell, and a stomatal apparatus of larger size and lower density on the leaf abaxial epidermis. These differences between *A. uljinensis* and *A. chinensis* have been summarized in Table 1.

**Table 1.** Morphological differences between *Astilbe uljinensis* and *Astilbe chinensis*.

Characters	Character state		*A. uljinensis*	*A. chinensis*
**Young rachis of rhizomatous leaf**				
Surface	color		always green	reddish to greenish brown
glandular hair	type	long hair	dense/whitish	sparse/whitish
		short hair	Absent	sparse/whitish
	length (mm)		0.6–1.0	less than 0.1 or 0.6–1.0
non-glandular hair	type	long hair	sparse/brownish	dense/whitish
**Mature inflorescence**				
glandular hair	type	long hair	dense/brownish	slightly dense/brownish
		short hair	Absent	slightly dense/brownish
	length (mm)		1.5–2.7	less than 0.2 or 1.0–2.2
**Leaf**				
terminal leaflet	shape		ovate-elliptic	rhomboid-elliptic
stomatal apparatus	length (µm)		23–26	20–24
	width (µm)		17–20	15–18
	density (no./1mm^2^)		28–51	51–66
adaxial epidermal cell	margin shape		weakly undulated	violently undulated
**Florescence**			late Jun. to early Aug.	late Jun. to early Sep.

#### Type.

**Figure 1.**
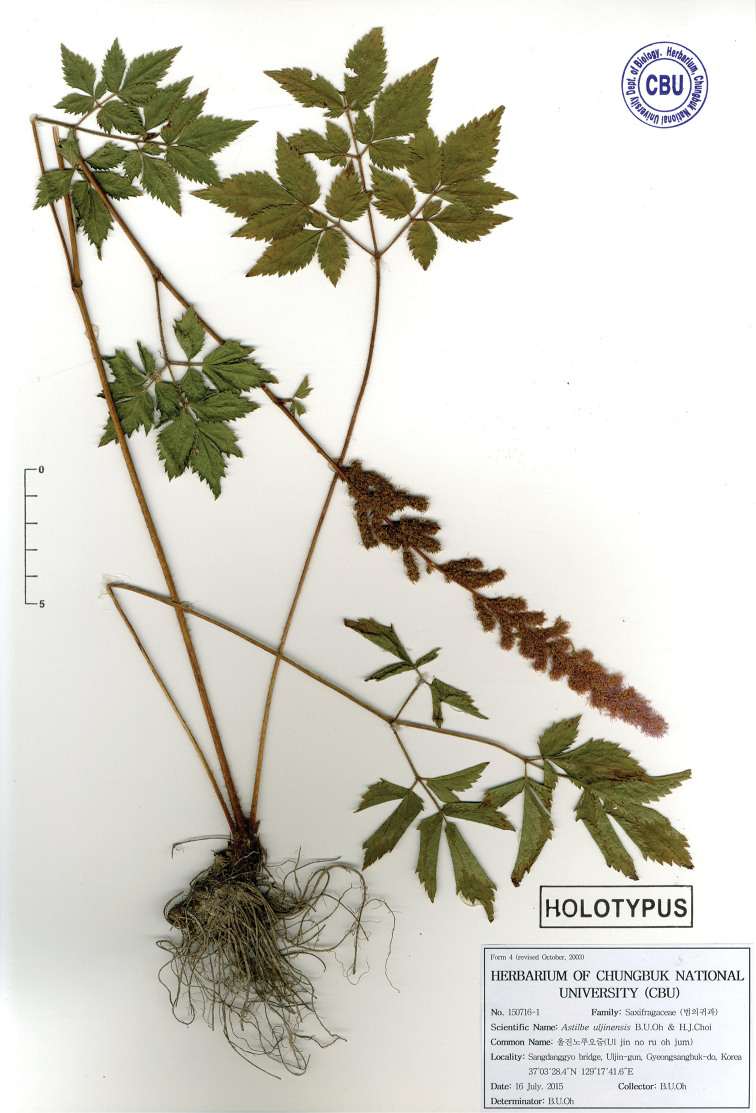
Holotype of *Astilbe uljinensis* B.U.Oh & H.J.Choi, *B.U.Oh et al*., *150716-1* (CBU).

South Korea. Gyeongsangbuk-do: Uljin-gun, Sangdanggyo bridge, sunny or shady forest in mountainous area, 110m, 37°03'28.4"N, 129°17'41.6"E, 16 July 2015, *B.U.Oh et al.*, *150716-1* (holotype: CBU!; isotype: CBU!) (Fig. 1).

#### Description.

Perennial, 57.4–125.7 cm tall. Roots fibrous, simple and stout, dark brown, 4.4–17.4 cm long, 0.2–0.4 mm in diam. Rhizomes extend, dark brown, 1.8–10.7 cm long, 12.6–26.8 mm in diam. Young shoots with dense long whitish glandular hair and sparse long brownish non-glandular hair on the surface, always green, 2.1–2.5 mm in diam. Rhizomatous leaves 2–3 ternate compound, 3–8 growing from rhizome, 31.5–68.5 cm long, 16.7–26.2 cm wide; terminal leaflet ovate to elliptic, apex acute to acuminate, base rounded or cuneate, margin double serrate, green; blade 52.2–81.3 mm long, 22.4–47.7 mm wide; rachis with dense long glandular hair. Bracts ternate leaf or 2–3 ternate compound, 0–2 growing from the rachis, usually smaller than rhizomatous leaves, 6.4–27.7 cm long, 3.6–13.7 cm wide. Inflorescence raches below bract erect, with dense glandular hair, green to reddish brown, 29.0–91.9 cm long, 2.3–4.7 mm in diam; those above bract developing panicle, 12.3–34.2 cm long, 0.8–2.2 mm in diam, with dense long brown glandular hair; peduncle, 32–40 from rachis, 2.2–7.4 cm long, 0.3–0.8 cm wide; pedicel 0.2–0.6 mm long. Flowers dense on peduncle, purplish red, 6.4–7.7 mm long, 1.4–2.2 mm wide; sepal 5, ovate, apex rounded, margin with short glandular hair, surface glabrous, white, 1.2–1.9 mm long, 0.8–1.0 mm wide; petal 5, linear, 1–veined, apex acute, 6.1–7.4 mm long, 0.2–0.3 mm wide; stamen 10, 3.2–3.9 mm long; filament linear, 2.8–3.4 mm long; anther ellipsoid, bright yellow to purple, 0.4–0.5 mm long; carpel 2, ovoid, surface glabrous, 2.0–2.3 mm long, 1.0–1.4 mm in diam. Fruits capsule, 2-parted, ovoid, green, surface glabrous, 3.3–3.8 mm long, 1.4–1.5 mm wide. Seeds numerous, irregularly ellipsoidal, dark brown, 1.0–1.4 mm long, 0.3–0.4 mm wide.

#### Distribution and habitat.

Currently, *A. uljinensis* is observed only in the central regions of South Korea, including Gangwon-do (Pyeongchang-gun, Samcheok-si, Gangneung-si) and Gyeongsangbuk-do (Uljin-gun). The species is found either in shady forests or on slopes near streams.

#### Phenology.

Flowering occurred between early June and late August. Fruiting occurred between July and October.

#### Conservation status.

At present, the known habitats of this new species are not being protected legally. In addition, since this species generally grows near streams, it is vulnerable to flooding. Previously, up to 10–20 individuals were found by the authors in some of this species’ populations. During the course of the present study, only a very small number of individuals were observed in some of the known habitats, probably indicating a decline in the plant population. However, the conservation status for the known populations has not been thoroughly investigated to date. Therefore, in accordance with the IUCN (2019) red list criteria, we suggest that this species should be treated as Data Deficient (DD).

#### Additional specimens examined.

South Korea. Gangwon-do: Pyeongchang-gun Odaesan, 4 July 1989, *B.H.Choi*, *4090* (KH!); Samcheok-si Deokhangsan, 16 July 2008, *K.O.Yoo*, *69542* (KWNU!); Samcheok-si Geombongsan, 18 July 2008, *K.O.Yoo*, *69544* (KWNU!); Samcheok-si Deokpung river, 11 July 2013, *B.U.Oh et al.*, *130711-4* (CBU!); Samcheok-si Sagok-ri, 11 July 2013, *S.H.Han & G.H.Kim*, *130711-3* (CBU!); Gangneung-si Sogeumgang, 24 June 1988, *C.M.Nam & H.C.Choi*, *7056* (KWNU!). Gyeongsangbuk-do: Uljin-gun Sangdanggyo bridge, 11 July 2013, *S.H.Han & G.H.Kim*, *130711-1* (CBU!); Uljin-gun Deokgugyohoe stream, 11 July 2013, *S.H.Han & G.H.Kim*, *130711-2* (CBU!).

**Figure 2.**
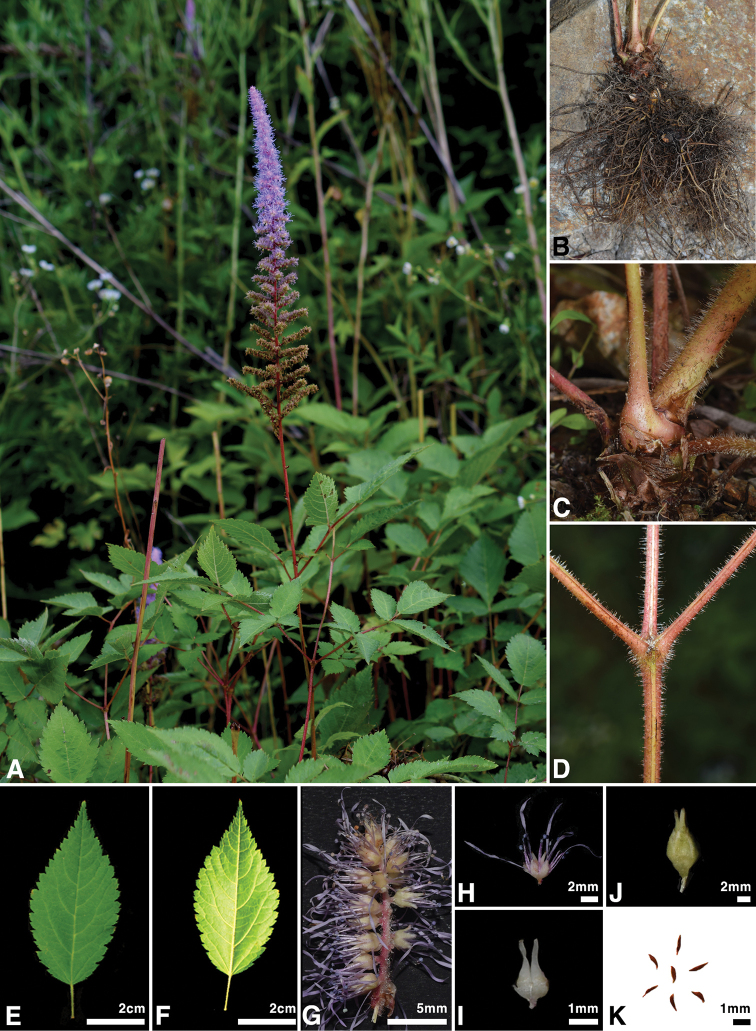
Morphological characteristics of *Astilbe uljinensis***A** habit **B** rhizome and root **C** base of rhizomatous leaf **D** secondary leaf axis **E** upper side of terminal leaflet **F** lower side of terminal leaflet **G** secondary rachis of inflorescence **H** flower **I** carpel **J** capsule **K** seed.

## Discussion

**Figure 3.**
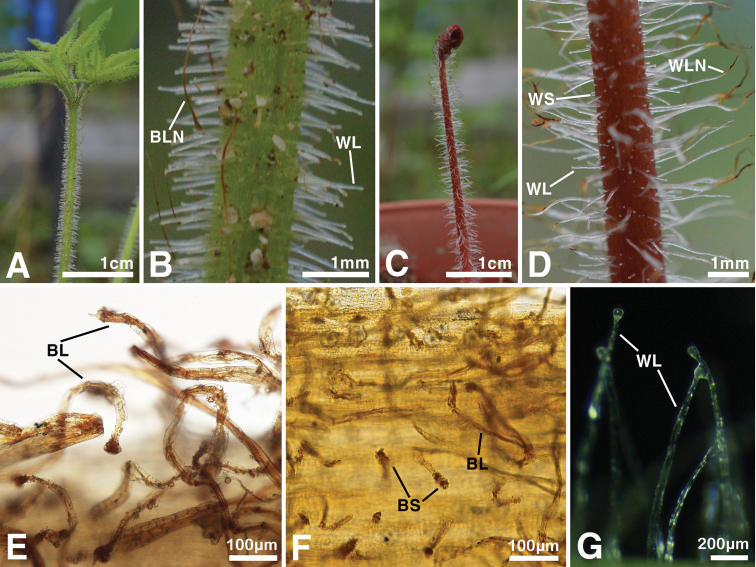
Shapes of trichomes on *Astilbe uljinensis* and *Astilbe chinensis***A, B, E, G***A. uljinensis***C, D, F***A. chinensis*. Abbreviations. BLN, Brownish Long Non-Glandular Hairs; WLN, Whitish Long Non-Glandular Hairs; WL, Whitish Long Glandular Hairs; WS, Whitish Short Glandular Hairs; BL, Brownish Long Glandular Hairs; BS, Brownish Short Glandular Hairs.

In the present study, the morphology of the trichomes on the plant body was studied extensively (Table 1; Fig. 3). When compared with the most similar *Astilbe* species, *A. chinensis*, this undescribed plant has only brownish long glandular hairs on its inflorescence (Fig. 3E), while *A. chinensis* has both brownish long glandular hairs and brownish short glandular hairs on it (Fig. 3F). In addition, the undescribed species has long whitish glandular hairs and long brownish non-glandular hairs on the young rachis of the rhizomatous leaves (Fig. 3B), while *A. chinensis* has short and long whitish glandular hairs, and long whitish non-glandular hairs on it (Fig. 3D).

**Figure 4.**
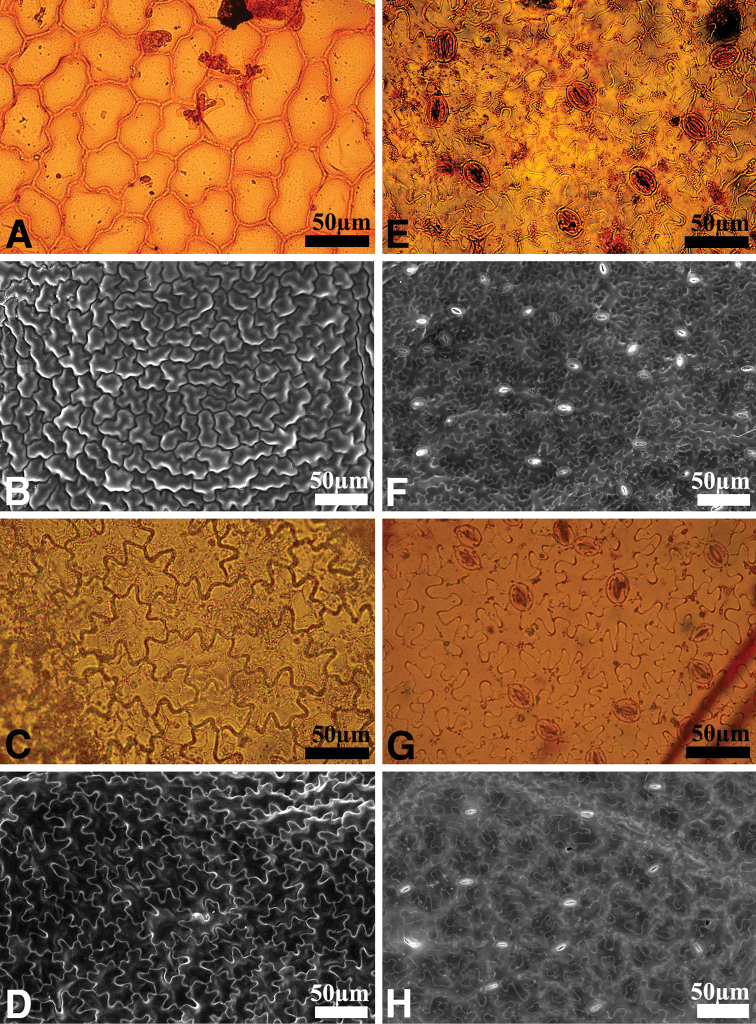
Differences in the leaf epidermis of *Astilbe uljinensis* and *Astilbe chinensis***A, B, E, F***A. uljinensis***C, D, G, H***A. chinensis*; Left: adaxial surface; Right: abaxial surface **A, C, E, G** Light Microscope image **B, D, F, H** by Scanning Electron Microscope image. Scale bars: 50 µm.

The shape of the leaf epidermal cells was also distinct in this undescribed plant. The adaxial epidermal cells of the new species had weakly undulated margins, while those of *A. chinensis* had violently undulated margins (Fig. 4). The stomatal apparatus on the abaxial leaf epidermis of the new species was slightly larger than that of *A. chinensis* (Table 1), and the density of the stomatal apparatus was lower in the new species compared to *A. chinensis* (Table 1). The shape of the terminal leaflet was ovate-elliptic in the new species (Fig. 2E, F), differing from the rhomboid-elliptic leaflet of *A. chinensis*.

The present study revealed that the morphology of the trichomes on the plant body, and their density, could be important characteristics for the identification of the newly described species, at the genus level. In addition, the geographical distribution of the undescribed plant is restricted to the central regions of South Korea, Gangwon-do and Gyeongsangbuk-do, which is also distinct compared to the distribution of other *Astilbe* species. From both the morphological observation and the recognized distribution patterns, we conclude that the previously undescribed plant should be treated as a new species. In this new species, both the macro-morphological and the micro-morphological characteristics are potentially important taxonomic keys for its identification.

## Supplementary Material

XML Treatment for Astilbe uljinensis
